# Supplementary material for: Infection and transmission of ancestral SARS-CoV-2 and its alpha variant in pregnant white-tailed deer
Source: Emerg Microbes Infect. 2021 Dec 21;11(1):95–112. doi: 10.1080/22221751.2021.2012528 (PMC8725908; doi:10.1080/22221751.2021.2012528)
Supplement: Supplemental Material [file TEMI_A_2012528_SM6231.docx]

**SUPPLEMENTARY FIGURES 1-5**

**
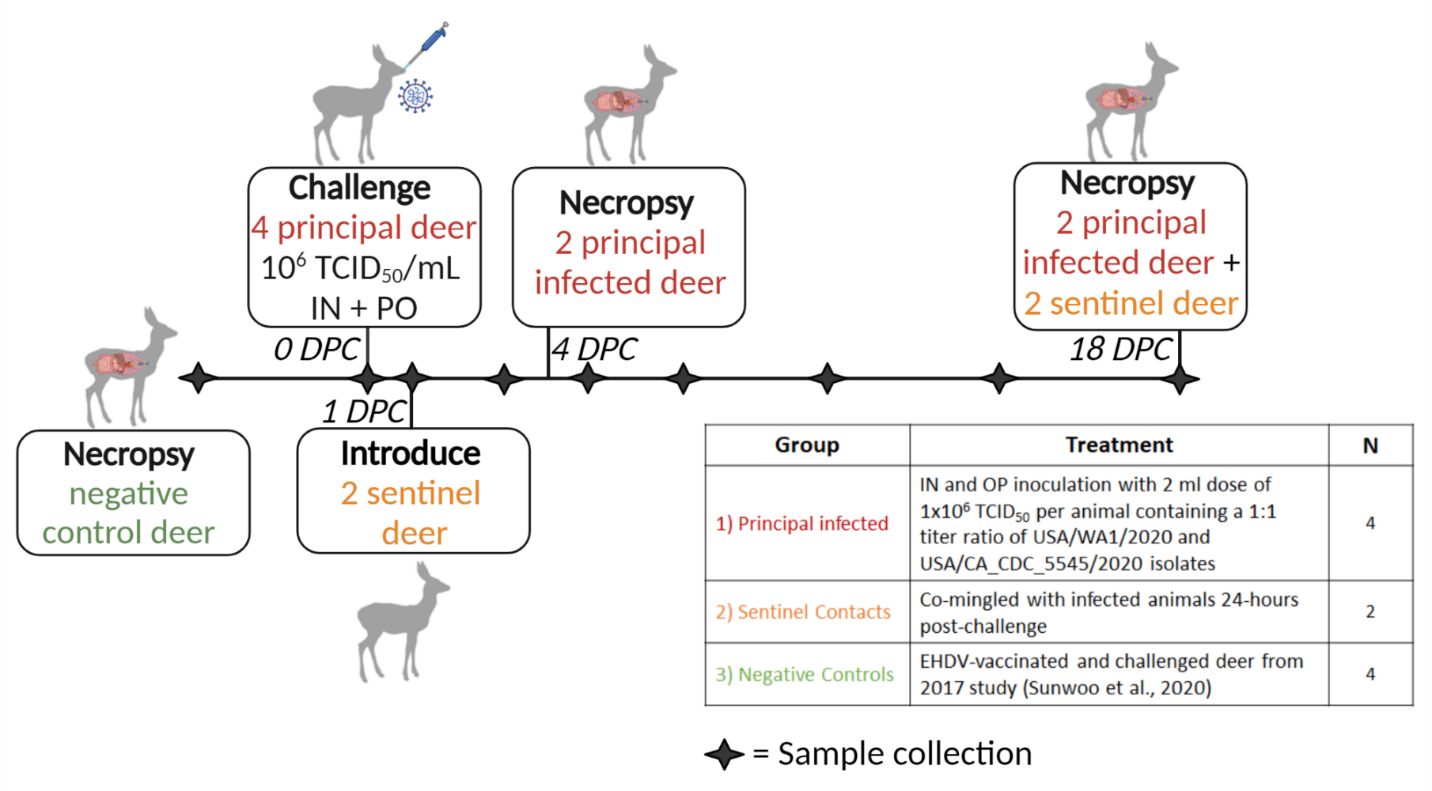
**

**Supplementary Figure 1. Experimental design.** Ten female white-tailed deer were split into three groups as follows: *i)* four principal infected deer, *ii)* two sentinel contact deer, and *iii)* four non-inoculated control deer. Group 1 was inoculated simultaneously via intra-nasal and oral routes with a 2 ml dose of 1x10^6^ TCID_50_ per animal containing an approximate 1:1 titer ratio of the lineage A WA1 strain and an alpha VOC B.1.1.7 strain of SARS-CoV-2. Group 2 deer (n=2) were used as sentinel contact animals and were not challenged directly. These sentinel deer were placed up air current of the room’s directional airflow and separated from the principal infected group by an 8-foot tall, solid partition wall on the day of challenge, provided separate food and water, and re-introduced to principal infected (group 1) 24-hours post infection. Nasal, oral, and rectal swabs were collected on days 0, 1, 3, 5, 7, 10, 14, and 18 post-challenge. Whole blood and serum were collected on 0, 3, 7, 10, 14, and 18 DPC. Two principal infected deer (group 1) were euthanized for *postmortem* examination on 4 days-post-challenge (DPC) to evaluate the acute phase of infection. The four remaining deer, consisting of two sentinels and two principal infected, were maintained for the duration of the 18-day study to evaluate contact transmission and the convalescent stage of infection. The control deer were part of a separate study (23).

**Supplementary Figure 2**. **Read coverage of sequenced samples from SARS-CoV-2 co-infected white-tailed deer.** Swab and tissue homogenate samples from white-tailed deer co-infected with the SARS-CoV-2/human/USA/WA1/2020 (lineage A WA1) and SARS-CoV-2/human/USA/CA-5574/2020 (alpha VOC B.1.1.7) strains were analyzed using next generation sequencing.

**Supplementary Figure 3. Daily Temperatures.** Rectal temperatures were taken from sedated deer on 0, 1, 3, 5, 7, 10, 14, 18 DPC.


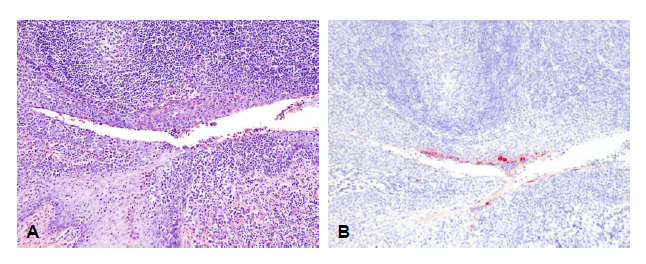


**Supplementary Figure 4. Histopathological lesions in the tonsil of a primary infected deer at 4 DPC.** Histologic alterations and SARS-CoV-2 antigen distribution in the tonsil of a white-tailed deer at 4 DPC. Foci along the lining epithelium are characterized by erosion and sloughing of superficial layers of the stratified squamous epithelium, with moderate numbers of transmigrating neutrophils and lymphocytes (**A**), and few superficial epithelial cells containing viral antigen (**B**). H&E and Fast Red, 100X total magnification.


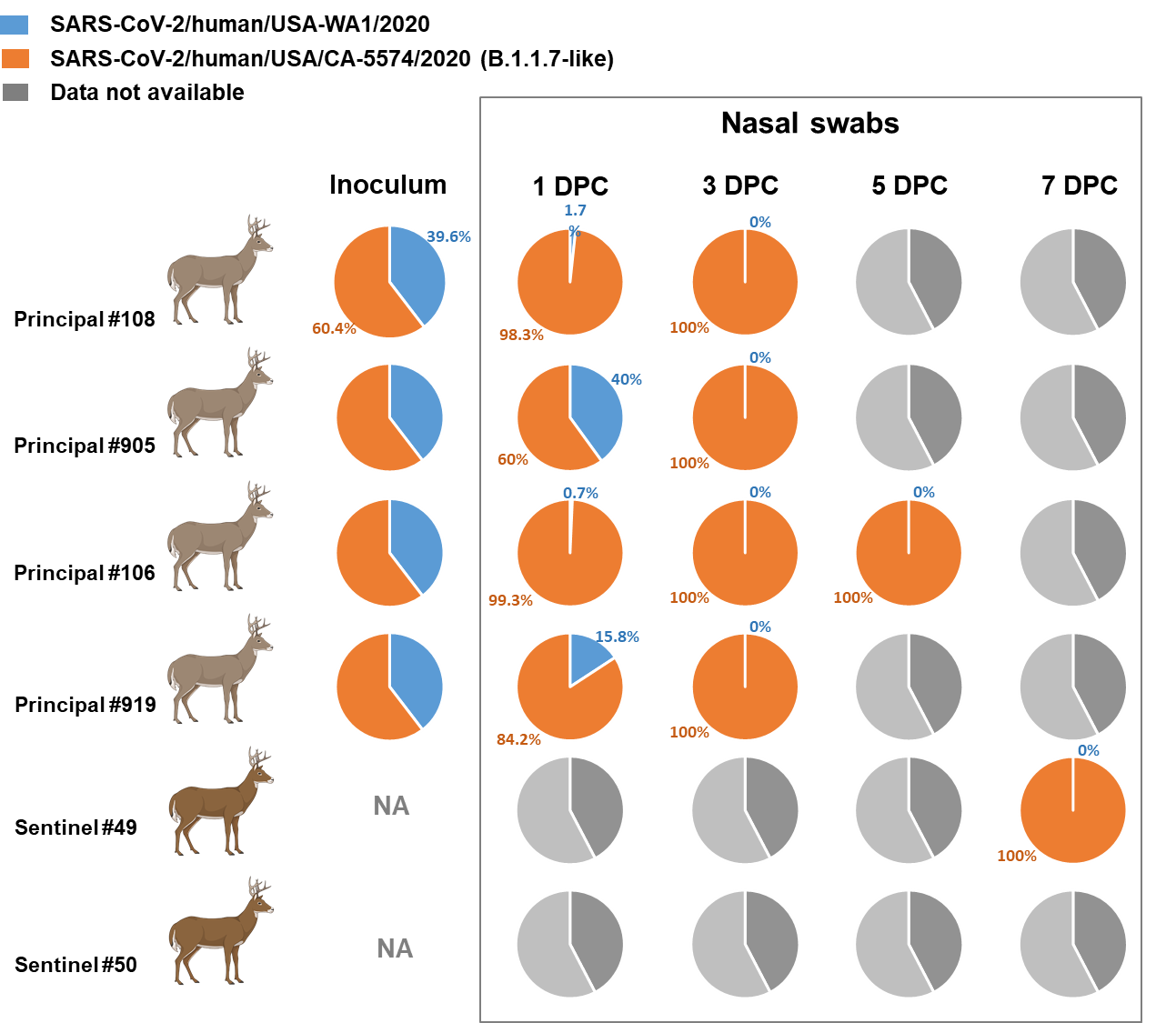


**Supplementary Figure 5. Next-generation sequencing of swabs collected from SARS-CoV-2 co-infected white-tailed deer.** cDNA products of SARS-CoV-2 RNA extracted from nasal swabs were sequenced on the Illumina NextSeq platform to evaluate the *in vivo* competition between the ancestral lineage A WA1 (SARS-CoV-2/human/USA/WA1/2020) and the alpha VOC B.1.1.7 (SARS-CoV-2/human/ USA/CA-5574/ 2020) strains.

**SUPPLEMENTARY TABLES – 1 & 2**

| **Supplementary Table 1. Presence of SARS-CoV-2 RNA (CN/mg) in fetuses and associated tissues.** | | | | | | |
| --- | --- | --- | --- | --- | --- | --- |
|  | Fetal lung | Fetal liver | Fetal kidney | Fetal spleen | Placenta | Uterus |
| *Necropsied 4 DPC* | | | | | | |
| #108; no fetus | NA | NA | NA | NA | NA | 1.47E+02* |
| #905, fetus 1 | ND | ND | ND | ND | ND | ND |
| #905, fetus 2 | 1.38E+02 | 5.89E+01* | ND | 2.55E+02 | 1.30E+02 | ND |
| #905, fetus 3 | ND | ND | ND | 1.22E+02* | ND | ND |
| *Necropsied 18 DPC* | | | | | | |
| #106, fetus 1 | ND | NC | NC | NC | NC | ND |
| #106, fetus 2 | ND | NC | NC | NC | NC | ND |
| #106, fetus 3 | ND | ND | NC | NC | NC | ND |
| #919, fetus 1 | ND | ND | NC | NC | NC | ND |
| #919, fetus 2 | ND | ND | ND | ND | NC | ND |
| #919, fetus 3 | NC | NC | NC | NC | NC | ND |
| #49, fetus 1 | NC | NC | NC | NC | NC | ND |
| #49, fetus 2 | NC | NC | NC | NC | NC | ND |
| #50, fetus 1 | ND | ND | NT | ND | ND | ND |
| NA=not applicable; ND=not detected; NC=not collected; NT=not tested; *=suspect positive | | | | | | |

| **Supplementary Table 2. Summary of histological and immunohistochemical (IHC) findings in the respiratory tract of SARS-CoV-2-infected white-tailed deer.** | | |
| --- | --- | --- |
| *Category (DPC)* | *Histological findings* | *IHC findings* |
| Principal (4 DPC) | Overall, changes were mild to moderate   - lymphohistiocytic and neutrophilic rhinitis - erosive to suppurative tracheitis - erosive bronchitis with mixed peribronchiolitis | - Infected respiratory epithelium of affected trachea and bronchi - Rare intrabronchiolar necrotic epithelial cells and leukocytes with viral antigen |
| Principal (18 DPC) | - Minimal lymphoplasmacytic tracheitis - Mild peribronchiolar/perivascular lymphocytic cuffing | No viral antigen detected |
| Sentinel (18 DPC) | - Mild to moderate lymphoplasmacytic and erosive tracheitis - Minimal peribronchiolar lymphocytic cuffing | No viral antigen detected |
